# Supplementary material for: X-Linked Epilepsies: A Narrative Review
Source: Int J Mol Sci. 2024 Apr 8;25(7):4110. doi: 10.3390/ijms25074110 (PMC11012983; doi:10.3390/ijms25074110)
Supplement: Supplementary file 1 [file ijms-25-04110-s001.zip › ijms-2935635-supplementary.pdf]

Supplementary Table S1

**Gene**	#patients	Phenotype
PCDH19	8 patients	Typical *PCDH19*-DEE with clusters of FS, focal S, hypomotor and affective seizures
CDKL5	3 patients harboring *de novo* variants<br><br>1 patient with Xp22 deletion (13.804 Kb) including *CDKL5*	Tonic-spasms during sleep<br><br>Drug-resistant epileptic encephalopathy (prevalent tonic seizures)
MECP2	72 patients	Both focal and generalized epilepsy (15/72 drug-resistant; about 21%)
MECP 2 duplication	2 males and 1 female	2 (1F, 1M) Lennox Gastaut Syndrome<br><br>1 M with Lennox-like phenotype (late-onset drug-resistant epileptic spasms)
FLNA	2 patients	Focal epilepsy, drug responsive
IQSEC2	1 patient	Generalized epilepsy (tonic-clonic and atonic seizures)
NEXMIF	2 patients	Epilepsy with eyelid myoclonia and Intellectual disability<br><br>Focal epilepsy with ID

Legend: DEE: developmental and epileptic encephalopathy; F: female; FS: febrile seizures; ID: intellectual disability; M: male.
